# Retrospective cohort trial protocol of screw fixation compared with hemiarthroplasty for displaced femoral neck fractures in elderly patients

**DOI:** 10.1097/MD.0000000000022397

**Published:** 2020-09-25

**Authors:** Boquan Qin, Linxian Cui, Yi Ren, Hui Zhang

**Affiliations:** a Department of Orthopaedic Surgery, West China Hospital, Sichuan University; b Department of Cardiology, Sichuan Provincial People's Hospital, University of Electronic Science and Technology of China, Sichuan, China.

**Keywords:** displaced femoral neck fractures, hemiarthroplasty, protocol, revision, screw fixation

## Abstract

**Background:**

There is limited evidence for the evaluation of the efficacy and safety of the hemiarthroplasty versus screw fixation in elderly patients with the displaced femoral neck fractures. Our current investigation aimed at assessing the complications, functional outcome, and revision rate of the patients (over 65 years old) who received internal fixation or hemiarthroplasty via a same senior surgeon.

**Methods:**

A retrospective study was conducted on elderly patients with displaced femoral neck fractures from May 2014 to February 2018. The current study was carried out at our hospital and it was approved through our institutional review committee of West China Hospital. Inclusion criteria were as follows: the patients were 65 years or older, this is the anesthesia grade. The higher grade of the patients,the greater risk of surgery. level I–III, and the patients with displaced intracapsular fractures of the femoral neck, with the radiographic and clinical follow-up of 12 months or more. The major outcome was the revision rate between the 2 groups. And the secondary outcomes contained the life quality and functional outcome detected via utilizing the interview-administered and self-administered questionnaires, length of hospital stay, surgery time, and hip-related complications (such as hip dislocation, loosening or breakage of implant, wound problems, infection, osteolysis, neurovascular injury, and bone nonunion).

**Results:**

It was assumed that hemiarthroplasty would result in fewer revisions or complications and better functional scores in comparison with internal fixation technique.

## 1 Introduction

Displaced femoral neck fractures are the most familiar injuries in the elderly. With the increase of the elderly population in China, the number of displaced femoral neck fractures is also increasing.^[1–3]^ They can be challenging to treat, as the femoral neck lacks periosteum and therefore relies on direct bone healing, resulting in the rates of osteonecrosis and nonunion of femoral head is high. Although the incidence of injury is high, in the elderly patients, the surgical treatment of displaced femoral neck fractures is still uncertain.^[4–8]^

Internal fixation is the optimism method to treat the elderly displaced femoral neck fractures patients. An alternative treatment option for the elderly patients with displaced femoral neck fractures is hemiarthroplasty, because the treatment allows the patient to move early.^[9]^ Nevertheless, the optimal treatment of a independent, removable patients with the femoral neck displaced intracapsular fracture is still controversial.^[10–14]^ Many former researches have conducted the comparison between internal fixation and hemiarthroplasty or either total hip arthroplasty, and the obtained results indicate that the internal fixation technique is less effective, and its reoperation rate is between 18% and 47%.^[15–20]^

Nevertheless, there is limited evidence for the evaluation of the efficacy and safety of the hemiarthroplasty versus screw fixation in elderly patients with the displaced femoral neck fractures. The superiority of the 2 approaches remains uncertain owing to the lack of a direct comparison of clinical effects between the 2 techniques in the present literature. Our current investigation aimed at assessing the complications, functional outcome, and revision rate of the patients (over 65 years old) who received internal fixation or hemiarthroplasty via a same senior surgeon. It was assumed that hemiarthroplasty would result in fewer revisions or complications and better functional scores in comparison with internal fixation technique.

## 2 Materials and methods

### 2.1 Study design

A retrospective study was conducted on elderly patients with displaced femoral neck fractures from May 2014 to February 2018. All cases were performed by a surgeon. The current study was carried out at our hospital and it was approved through our institutional review committee of West China Hospital (SC001090). This current investigation also has been registered in Research Registry (researchregistry5956).

### 2.2 Study population

Inclusion criteria were as follows: the patients were 65 years or older, with the American Society of Anesthesiologists level I–III, and the patients with displaced intracapsular fractures of the femoral neck, with the radiographic and clinical follow-up of 12 months or more. And exclusion criteria contained the patients under the age of 65, preexisting hip abnormality (such as osteoarthritis), patients with the American Society of Anesthesiologists IV level, pathological fracture, and patients with physical or medical comorbidities that restricting walking, and patients with ongoing infectious disease.

### 2.3 Intervention and techniques

All the surgeries in our current study were implemented via a senior surgeons, or via a researcher under his direct supervision. Prior to incision, each patient was given the general anesthesia to prepare for the operation of hip joint, and then covered with a routine sterile manner. Here are the technical details of 2 groups:

#### 2.3.1 Hemiarthroplasty group

All the operations were conducted through the fellowship trained consultants with a posterior or lateral approach depending on the surgeon's preference. The standard 130 mm cementless porous coated femoral stems were utilized. The head size of the prosthesis is increased by 2 mm, which could accurately replicate the femoral head of the patient, and the femoral head size was determined using the hemispherical template during surgery.

#### 2.3.2 Screw fixation group

Two 7.0 mm diameter partially threaded, cannulated, and cancellous screws (Hip Pins; Smith & Nephew) were inserted, with inferior screw as close to the medial cortex as possible. The 2 screws were positioned posteriorly and centrally and then they inserted as deep as possible in order to ensure that the screws can be fixed in subchondral bone. And if femoral head tilts backwards, the surgeon will attempt a closed reduction.

### 2.4 Postoperative care

One day after operation, a medium volume hemovac drain was placed and then removed. Low-dose warfarin or aspirin were utilized to prevent thrombosis after operation. The patients took 5 mg of warfarin orally on the night of operation, and then adjusted daily as needed to keep the international standardized ratio of 1.8 to 2.2. All patients were given a same standardized multimodality pain regimen after operation, with 2 doses of 200 mg of celecoxib, 4 doses (1 g) of the acetaminophen, the morphine (first 48 hours) or the tramadol (after 48 hours) to relieve pain. All the patients adopted a same rehabilitation program after operation. On the first day after surgery, they received exercise training in the range of activities and used crutches to carry out the partial weight-bearing. At the same time, all the patients were given a same preoperative antibiotic regimen.

### 2.5 Outcome measures

The major outcome was the revision rate between the 2 groups. And the secondary outcomes contained the life quality and functional outcome detected via utilizing the interview-administered and self-administered questionnaires, length of hospital stay, surgery time, and hip-related complications (such as hip dislocation, loosening or breakage of implant, wound problems, infection, osteolysis, neurovascular injury, and bone nonunion). Functional outcome questionnaires include hip disability and osteoarthritis outcome score (HOOS) and the Harris hip score (HHS) (Table 1).

**Table 1:**
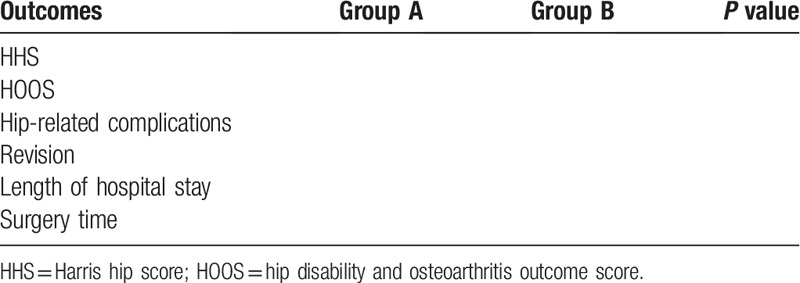
Postoperative outcomes.

HHS was developed to evacuate the hip surgery outcomes and to assess a variety of hip disabilities and the related treatments in adult population. The areas covered include function, pain, the range of motion, and loss of deformity. The highest score is 100 points (optimism possible outcome), including function (7 items, between 0 and 47 points), pain (1 item, between 0 and 44 points), the motion range (2 items, 5 points), and the loss of deformity (1 item, 4 points).

HOOS is a tool to evaluate the patients’ views on the hip and the related issues. It is designed to be utilized in adults with the hip disability without or with osteoarthritis. HOOS was composed of 5 subscales: pain, function of daily activities, other symptoms, function of recreation and exercise, and quality of life associated with hip joints.

### 2.6 Statistical analysis

All the analyses were implemented with the software of SAS (version 9.3 and SAS Enterprise Guide 6.1; SAS Institute, American NCSU). The error probability of type-I of all the analysis was set as *P* < .05. The medians and proportions were utilized to describe the baseline cohort characteristics, and then the were compared baseline cohort characteristics via applying chi-square tests for the categorical variables and Wilcoxon rank sum tests for the continuous variables between the 2 groups. For all the tests, *P* < .05 was considered as significance in statistics.

## 3 Discussion

Intracapsular hip fracture is a most familiar reasons for elderly patients to enter the acute orthopedic ward. In the absence of displacement, almost all the intramedullary femoral neck fractures in China have been treated with the screw osteosynthesis, there is no consensus on the displaced femoral neck fractures treatment. Our present investigation aims at assessing the complications, functional outcome, and revision rate of the patients (over 65 years old) who received internal fixation or hemiarthroplasty via a same senior surgeon. It is assumed that hemiarthroplasty would result in fewer revisions or complications and better functional scores in comparison with internal fixation technique. The limitations of our research involved the use of a single surgeon, a single implant model and manufacturer, and the lack of patient randomization, as well as the lack of advanced imaging techniques (computed tomography) for the accurate measurements before and after the measurements. Furthermore, the limitations of our present investigation contain the inherent limitations in any retrospective cohort research, involving the observational bias or the possibility of selection.

## Author contributions

**Conceptualization:** Boquan Qin, Yi Ren, Hui Zhang.

**Data curation:** Boquan Qin, Linxian Cui, Yi Ren.

**Formal analysis:** Boquan Qin, Linxian Cui, Yi Ren.

**Funding acquisition:** Hui Zhang.

**Investigation:** Boquan Qin, Yi Ren.

**Methodology:** Boquan Qin, Linxian Cui.

**Resources:** Hui Zhang.

**Software:** Linxian Cui.

**Supervision:** Hui Zhang.

**Validation:** Linxian Cui, Yi Ren.

**Visualization:** Linxian Cui, Yi Ren.

**Writing – original draft:** Boquan Qin.

**Writing – review & editing:** Hui Zhang, Linxian Cui.
